# Human Adipose-Derived Stem Cells Exhibit Enhanced Proliferative Capacity and Retain Multipotency Longer than Donor-Matched Bone Marrow Mesenchymal Stem Cells during Expansion In Vitro

**DOI:** 10.1155/2017/2541275

**Published:** 2017-05-03

**Authors:** Kimberley L. Burrow, Judith A. Hoyland, Stephen M. Richardson

**Affiliations:** ^1^Division of Cell Matrix Biology and Regenerative Medicine, School of Biological Sciences, Faculty of Biology, Medicine and Health, The University of Manchester, Stopford Building, Oxford Road, Manchester M13 9PT, UK; ^2^NIHR Manchester Musculoskeletal Biomedical Research Unit, Central Manchester Foundation Trust, Manchester Academic Health Science Centre, Manchester, UK

## Abstract

Bone marrow-derived mesenchymal stem cells (MSCs) and adipose-derived multipotent/mesenchymal stem cells (ASCs) have been proposed as the ideal cell types for a range of musculoskeletal tissue engineering and regenerative medicine therapies. However, extensive in vitro expansion is required to generate sufficient cells for clinical application and previous studies have demonstrated differences in the proliferative capacity and the impact of expansion on differentiation capacity of both MSCs and ASCs. Significantly, these studies routinely use cells from different donors, making direct comparisons difficult. Importantly, this study directly compared the proliferative capacity and multipotency of human MSCs and ASCs from the same donors to determine how each cell type was affected by in vitro expansion. The study identified that ASCs were able to proliferate faster and undergo greater population doublings than donor-matched MSCs and that senescence was primarily driven via telomere shortening and upregulation of p16^ink4a^. Both donor-matched MSCs and ASCs were capable of trilineage differentiation early in cultures; however, while differentiation capacity diminished with time in culture, ASCs retained enhanced capacity compared to MSCs. These findings suggest that ASCs may be the most appropriate cell type for musculoskeletal tissue engineering and regenerative medicine therapies due to their enhanced in vitro expansion capacity and limited loss of differentiation potential.

## 1. Introduction

Since their first isolation from bone marrow almost 50 years ago, mesenchymal stem cells (MSCs) have been extensively investigated and, due to their rapid proliferation and multipotent differentiation potential, MSCs have been proposed as ideal cells for tissue engineering and regenerative medicine therapies [1–6]. In particular, their ability to differentiate along the osteogenic, chondrogenic, and adipogenic lineages means they are currently being trialled in numerous orthopaedic applications to treat a range of degenerative conditions including osteoarthritis, congenital disorders (e.g., osteogenesis imperfecta), and defects such as nonunion fractures or critical-size bone defects. However, such applications require large numbers of cells, and thus, in vitro expansion of cells is essential prior to reimplantation. Studies on MSCs have suggested that expansion may be detrimental to subsequent differentiation potential, especially when cells undergo cellular senescence, and thus, there is a clinical imperative to ensure that functional expanded cells are used in such therapies to ensure appropriate outcomes.

Since MSCs were identified in bone marrow, multipotent stem cells have been isolated from other tissues, with adipose tissue identified as a tissue with a relatively high abundance of stem cells, even compared to bone marrow [7, 8]. These adipose-derived multipotent/mesenchymal stem cells (ASCs) appear to share similar morphologic and phenotypic characteristics with MSCs, including equivalent spindle-shaped morphology and similar expression of cell surface markers such as CD73, CD90, and CD105, albeit with evidence of additional markers such as CD34 on ASCs [9, 10]. Previous studies into MSC and ASC expansion suggest that ASCs retain morphological and genetic stability longer during expansion than MSCs and that they have a higher proliferative capacity and lower levels of cellular senescence compared to MSCs [11–16]. The original studies into ASCs also demonstrated similar multilineage differentiation potential to MSCs, although subsequent studies suggest that ASCs may have enhanced, equivalent, or diminished osteogenic [17–20], chondrogenic [18, 21–25], and adipogenic [19, 23, 24, 26, 27] potential compared to MSCs [28]. Similar controversy surrounds the extent to which in vitro expansion impacts differentiation capacity of MSCs and ASCs, and while the majority of studies demonstrate a decline in differentiation capacity in one or both cell types with time in culture, other studies suggest a preservation of differentiation capacity with in vitro expansion [14, 23, 29–38].

Crucially, however, while numerous investigations have compared MSCs and ASCs, in the majority of studies, cells are not derived from the same donor, meaning direct comparison between cell types is difficult, even in age-matched donors. Furthermore, where studies on donor-matched MSCs and ASCs have been conducted, they routinely focus only on differentiation potential in early passage cells, or where a comparison of ageing is made, and differentiation is assessed only along a limited number of lineages [21–23, 39]. This therefore makes it difficult to accurately assess the impact of in vitro expansion on MSC and ASC differentiation.

Functional comparisons of donor-matched MSC and ASC samples are essential to establish whether one or another tissue source may be more appropriate for application in musculoskeletal tissue engineering and regenerative medicine therapies. Furthermore, since such applications are likely to require in vitro expansion, it is essential to establish how extended culture impacts function of donor-matched MSC and ASC.

We hypothesised that donor-matched MSCs and ASCs would demonstrate differences in proliferative capacity and trilineage differentiation potential, thus making one cell type more appropriate for application in musculoskeletal tissue engineering and regenerative medicine therapies. Therefore, this study aimed to investigate how extended in vitro expansion of donor-matched human MSCs and ASCs affects their proliferation, senescence marker expression, and differentiation potential. To achieve this, cells were assessed at varying points throughout their in vitro lifespan, with findings providing important information that can help inform clinical decision making when identifying the most appropriate stem cell source for tissue engineering or regenerative medicine therapy where large numbers of cells are required for differentiation and implantation.

## 2. Methods

### 2.1. Isolation and Culture of MSCs and ASCs

All procedures and experiments were performed with relevant National Research Ethics Service and University of Manchester approvals. Bone marrow and subcutaneous adipose tissue samples were obtained from 5 donors (3 males, 2 females, mean age 44 years, range 28–55 years) undergoing hip replacement surgery who provided written informed consent.

Bone marrow aspirates were washed with PBS and then centrifuged to obtain a cell pellet, and the pellet was resuspended in Minimum Essential Medium *α*-modification (*α*MEM) (Sigma-Aldrich) which included nonessential amino acids, 110 mg/l sodium pyruvate, 1000 mg/l glucose supplemented with final concentrations of 100 U/ml penicillin, 100 *μ*g/ml streptomycin and 0.25 *μ*g/ml amphotericin, 2 mM GlutaMAX (Life Technologies), and 20% (*v*/*v*) FBS. MSCs were isolated using density gradient centrifugation as previously described [40]. Adipose tissue was finely minced with a scalpel and then ASCs were isolated enzymatically as previously described [41]. Both cell types were maintained at 37°C in a humidified atmosphere containing 5% (*v*/*v*) CO_2_; the culture medium was changed after 5 days and cells cultured to ∼80% confluence in *α*MEM as above supplemented with 10% FBS.

Expanded cells were used to assess the impact of long-term culture on population kinetics, telomere length, and multilineage differentiation potential along the chondrogenic, osteogenic, and adipogenic lineages.

### 2.2. Long-Term Culture and Population Kinetics

MSCs and ASCs were harvested after reaching ∼80% confluence using trypsin and propagated at 3000 cells/cm^2^, continuously until the time to perform 1 population doubling (PD) exceeded the initial PD time by 5. PD of cultured cells was calculated at every passage according to the equation LOG(final cell count/seeding density)/LOG2. Cumulative PDs were determined by successive addition of PD numbers generated each passage [42]. This data was used to estimate the percentage lifespan of cells at each passage, with <30% being defined as early lifespan complete (defined hereafter as ELSC), 50–60% being mid lifespan complete (MLSC), and >80% being defined as late lifespan complete (LLSC). These were used as timepoints in subsequent experiments.

### 2.3. Flow Cytometric Analysis of CD Profile

MSCs and ASCs were trypsinized at the end of the first passage, pelleted and resuspended in 1 ml cold FACS buffer (PBS solution supplemented with 0.5% bovine serum albumin (BSA) and 2 mM EDTA), and filtered through a premoistened 40 *μ*m cell strainer. Cells were then labelled using an MSC phenotyping kit (Miltenyi Biotec) according to the manufacturer's instructions. The kit contains fluorochrome-conjugated antibodies for CD73-APC, CD90-FITC, and CD105-PE, along with a cocktail of negative markers (CD14, CD20, CD34, and CD45) all conjugated to PerCP. Relevant IgG controls were also prepared and all samples analysed on a CyAn ADP flow cytometer (Beckman Coulter), with 10,000 events being recorded for each sample. Initial analysis was performed using Summit 4.3 software, with spectral overlap of fluorochrome emissions corrected through compensation using single-stained controls for each fluorochrome. Regions and gates were created so only single live cells were included in the analysis. Further analysis was performed using FlowJo.

### 2.4. Telomere Length Measurement Assay

DNA was extracted from MSCs and ASCs at ELSC, MLSC, and LLSC, using a QIAamp® DNA mini kit (Qiagen). Telomere restriction fragment length (TRFL) was determined using Southern blot analysis. DNA was digested using HinfI and RsaI enzymes and the digested DNA separated on 0.8% agarose gels. Gels were denatured and blotted overnight onto a positively charged nylon membrane. Hybridization with a digoxigenin- (DIG-) labelled telomere-specific probe was conducted using the DIG assay kit (Roche). After hybridization, membranes were washed and incubated with a DIG-specific antibody covalently coupled to alkaline phosphatase. Results were visualised using a chemiluminescent substrate and exposing the hybridization membrane to a chemiluminescent detection film. The films were then scanned and the mean TRFL calculated.

### 2.5. Assessment of Chondrogenic Differentiation Potential

Chondrogenesis was induced in MSCs and ASCs at ELSC, MLSC, and LLSC using 3D pellet cultures containing 2.5 × 10^5^ cells cultured in AQmedia high-glucose DMEM (Sigma) supplemented with 1% FBS, antibiotics, 1 mM sodium pyruvate, 100 *μ*M L-ascorbic acid 2-phosphate, 1 × 10^−7^ M dexamethasone, 1.25 mg/ml BSA, 40 *μ*g/ml L-proline, 5.4 *μ*g/ml linoleic acid, 1X ITS-X (Life Technologies), and 10 ng/ml TGF-*β*3. Media were replaced every 3 days for up to 21 days.

Expression of chondrogenic marker genes SOX-9, COL2A1, ACAN, and COL10A1 was assessed using quantitative real-time PCR (qPCR). Sulphated GAG accumulation was quantified using a sulphated glycosaminoglycan assay kit (Biocolor) after overnight digestion at 65°C with 125 *μ*g/ml papain, 200 mM sodium phosphate buffer at pH 6.4, 100 mM sodium acetate, 10 mM EDTA, and 5 mM L-cysteine hydrochloride. Absorption was measured at 650 nm and quantified against chondroitin sulphate reference standards. GAG concentration was normalised against DNA concentration quantified using the Quant-IT PicoGreen dsDNA assay kit (Life Technologies). To assess extracellular matrix deposition, pellets were fixed in PFA, embedded in paraffin, and then cut into 4 *μ*M sections. Sections were then stained with safranin O for identification of GAG deposition and stained with Masson's trichrome for identification of collagen. Sections were also assessed for aggrecan and type II collagen through immunohistochemistry using mouse anti-human monoclonal antibodies (AbDSeroTec and Developmental Studies Hybridoma Bank, resp.) and a secondary anti-mouse antibody (Santa Cruz Biotechnology) with detection by diaminobenzidine tetrahydrochloride (DAB).

### 2.6. Assessment of Osteogenic Differentiation Potential

MSCs and ASCs at ELSC, MLSC, and LLSC were cultured in monolayer at a density of 3 × 10^3^ cells/cm^2^ in osteogenic induction media containing *α*MEM supplemented with antibiotics, 10% FBS, 1 mM sodium pyruvate, 2 mM Glutamax (Life Technologies), 100 *μ*M L-ascorbic acid 2-phosphate, 1 × 10^−7^ M dexamethasone, and 10 mM *β*-glycerophosphate, with media changed every 3 days for up to 21 days. Expression of osteogenic marker genes RUNX2, alkaline phosphatase (ALPL), and osteocalcin (OCN) was determined using qPCR. Alizarin red staining was used to identify mineralisation deposits in the cell culture both qualitatively and quantitatively. ALP activity was quantified against a standard curve of 4-nitrophenol solution (Sigma-Aldrich) and expressed as *μ*mol 4-nitrophenol per ng/*μ*l DNA.

### 2.7. Assessment of Adipogenic Differentiation Potential

MSCs and ASCs at ELSC, MLSC, and LLSC were cultured in monolayer at a density of 3 × 10^3^ cells/cm^2^ and adipogenic induction media containing *α*MEM supplemented with 10% FBS, antibiotics, 2 mM Glutamax, 1 × 10^−8^ M dexamethasone, 250 *μ*M IBMX, and 66 nM insulin, with media changed every 3 days for up to 21 days. Expression of adipogenic marker genes PPAR*γ*, adipsin, LPL, and FABP4 was assessed using qPCR. The presence of lipid droplets were identified and quantified by staining with oil red O.

### 2.8. RNA Isolation, cDNA Synthesis, and qPCR

Total RNA was extracted using TRIzol reagent (Life Technologies) in combination with 1-bromo-3-chloro-propane (BCP) and isopropanol, with the addition of molecular grinding resin and high salt precipitation solution when extracting RNA from pellet cultures. cDNA was prepared using a high-capacity cDNA reverse transcription kit with RNase inhibitor (Life Technologies) and 600 ng of sample RNA. Reaction mixtures for amplification contained 10 ng of cDNA (2 *μ*l), 5 *μ*l 2X LuminoCt® qPCR ReadyMix (Sigma-Aldrich), 1 *μ*l forward primer, 1 *μ*l reverse primer, 0.5 *μ*l 6-FAM/BHQ1 probe (250 nM final concentration; Sigma-Aldrich), and 0.5 *μ*l 40x ROX internal reference dye. The primer sequences and concentrations are displayed in Table 1. All reactions were performed in triplicate using an ABI StepOnePlus Real-Time PCR System (Applied Biosystems), under standard conditions. Relative expression was normalised to the mean of the reference genes MRPL19 and EIF2B1, using the 2^−ΔCt^ method.

### 2.9. Statistical Analysis

Statistical analyses were performed using SPSS v13.0 or GraphPad Prism software. Data were assessed using intraclass correlation coefficient (ICC), a one-way analysis of variance (ANOVA), Spearman's rank correlation coefficient, and Mann-Whitney with significance defined as *P* < 0.05.

## 3. Results

### 3.1. Effect of In Vitro Expansion on CD Profile, Morphology, Population Doubling Number, and Telomere Length

**Figure 1:**
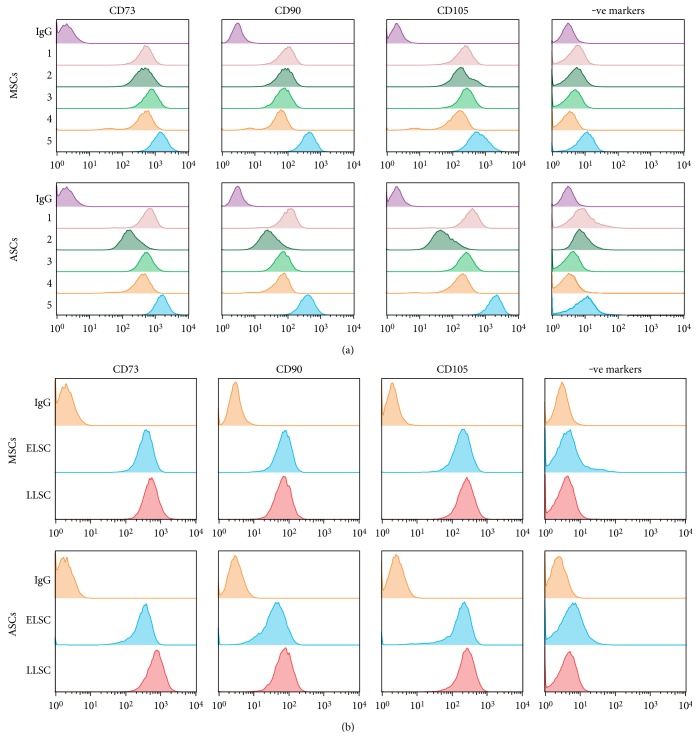
Assessment of MSC and ASC CD profile. (a) Expression of positive markers CD73, CD90, and CD105 was assessed using flow cytometry, along with expression of a panel of negative markers (CD14, CD20, CD34, and CD45) in donor-matched MSC and ASC samples (*n* = 5). Expression was compared to IgG controls. MSC and ASC numbers 1–5 represent donor-matched samples. (b) Expression of CD markers was further compared between ELSC and LLSC with no significant difference in expression identified with time in culture. Plots show representative data from one donor-matched sample and ELSC and LLSC compared to an IgG control.

The CD profile of MSCs and ASCs was comparable at ELSC (Figure 1(a)), with an average of >95% of both cells expressing the positive markers CD73, CD90, and CD105. The both cells demonstrated low levels of the negative marker panel (CD14, CD20, CD34, and CD45). CD marker profile did not change for any of the samples analysed with time in culture demonstrating stability of CD profile throughout their lifespan (Figure 1(b)).

**Figure 2:**
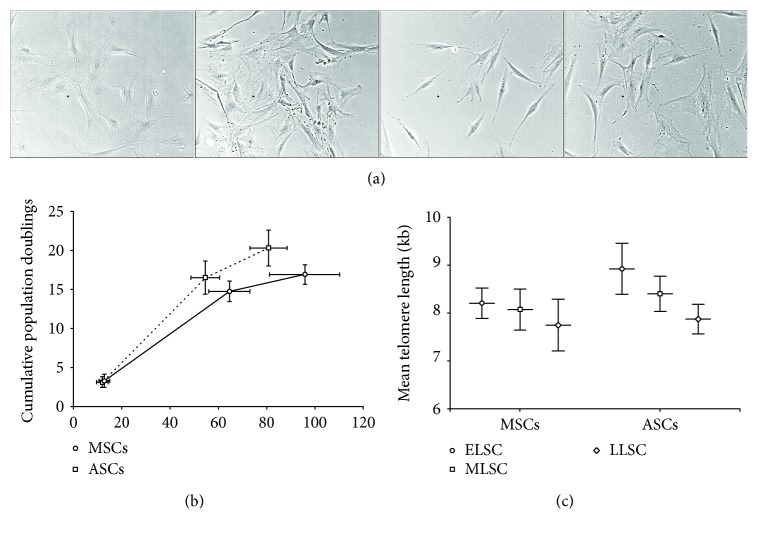
Population kinetics. (a) Representative phase-contrast microscopy images of donor-matched MSCs and ASCs during monolayer culture at ELSC and LLSC. (b) Cumulative population doublings over time in culture demonstrating enhanced proliferation rate in ASCs compared to donor-matched MSCs (*N* = 5). Points represent ELSC, MLSC, and LLSC, ±SEM for both CPD and days in culture. (c) Mean telomere length in donor-matched MSCs and ASCs at ELSC, MLSC, and LLSC (±SEM) demonstrating longer telomeres in ASCs compared to MSCs and a decrease in mean telomere length with time in culture in both cell types (*N* = 5). ∗ represents *P* < 0.05 at MLSC or LLSC compared to ELSC. + represents *P* < 0.05 in ASCs compared to MSCs at the same LSC.

Morphologically, both MSCs and ASCs displayed a characteristic spindle-shaped fibroblastic appearance during early lifespan in culture. By late lifespan, the majority of both MSCs and ASCs displayed a large, flattened polygonal morphology and ragged appearance, with the presence of stress fibres within the cytoplasm (Figure 2(a)), indicative of senescence.

Assessment of cumulative population doublings (CPD) over time in culture demonstrated similar rates of expansion between MSCs and ASCs at ELSC. However, assessment of CPD at MLSC and LLSC demonstrated greater PD number achieved by ASCs (MLSC: 16.5 ± 2.1 CPD; LLSC: 20.3 ± 2.3 CPD) compared with MSCs (MLSC: 14.7 ± 1.3 CPD; LLSC: 16.9 ± 1.2 CPD). In addition, the MLSC and LLSC timepoints for ASCs (MLSC: 54.6 ± 5.9 days; LLSC: 80.8 ± 7.7 days) were, on average, reached within a lower number of days in culture compared to MSCs (MLSC: 64.6 ± 8.5 days; LLSC: 95.8 ± 14.5 days) (Figure 2(b)).

Telomere length was measured on genomic DNA extracted from MSCs and ASCs at ELSC, MLSC, and LLSC (Figure 2(c)). Telomere length was significantly longer in ASCs compared to MSCs at every lifespan point. While MSCs did not show a significant decrease in telomere length with time in culture, ASCs demonstrated a significant decrease in mean telomere length between ELSC and LLSC and MLSC and LLSC.

**Figure 3:**
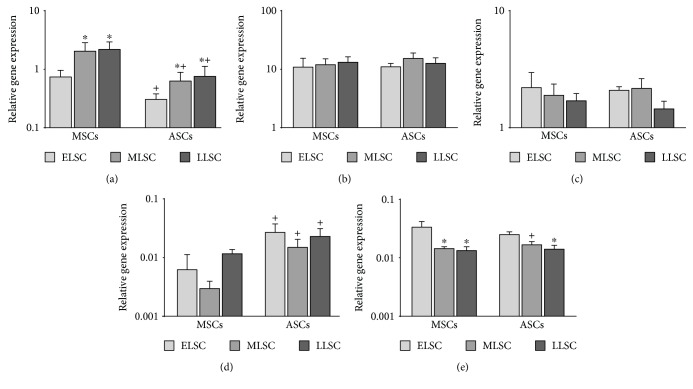
qPCR analysis of senescence marker genes p16^ink4a^ (a), p21 (b), and p53 (c) and pluripotency marker genes Nanog (d) and Oct-4 (e) in donor-matched MSCs and ASC at ELSC, MLSC, and LLSC. Data presented and relative gene expression normalised to the mean of the reference genes MRPL19 and EIF2B1 and plotted on a log scale (*N* = 5). Values are mean ± SEM. ∗ represents *P* < 0.05 at MLSC or LLSC compared to ELSC. + represents *P* < 0.05 in ASCs compared to MSCs at the same LSC.

Expression of senescence (p16^ink4a^, p21, and p53) and pluripotency (Nanog and Oct-4) related genes were detected using qPCR in MSCs and ASCs at ELSC, MLSC, and LLSC (Figure 3()). The data demonstrated that, of the senescence markers, p16^ink4a^ expression increased with advancing lifespan in culture for both MSCs and ASCs, with expression levels at MLSC and LLSC significantly raised compared to ELSC (Figure 3(a)). Expression of p16^ink4a^ was significantly lower in ASCs at all timepoints compared to MSCs. Senescent markers p21 and p53 showed no significant change in expression with time in culture for either MSCs or ASCs and no significant difference in expression levels between tissue source of cells (Figures 3(b) and 3(c)). Pluripotency marker Nanog showed no significant change in expression level during prolonged culture in either MSCs or ASCs, although a significantly higher level of expression was present in ASCs compared with MSCs at each timepoint assessed (Figure 3(d)). Conversely, no significant difference in the expression levels of pluripotency marker Oct-4 was seen between MSCs and ASCs during prolonged culture; however, a significant decrease in Oct-4 expression was identified at both MLSC and LLSC compared to ELSC for cells from both tissue sources (Figure 3(e)).

### 3.2. Effect of In Vitro Expansion on Differentiation Potential

#### 3.2.1. Chondrogenic Differentiation

**Figure 4:**
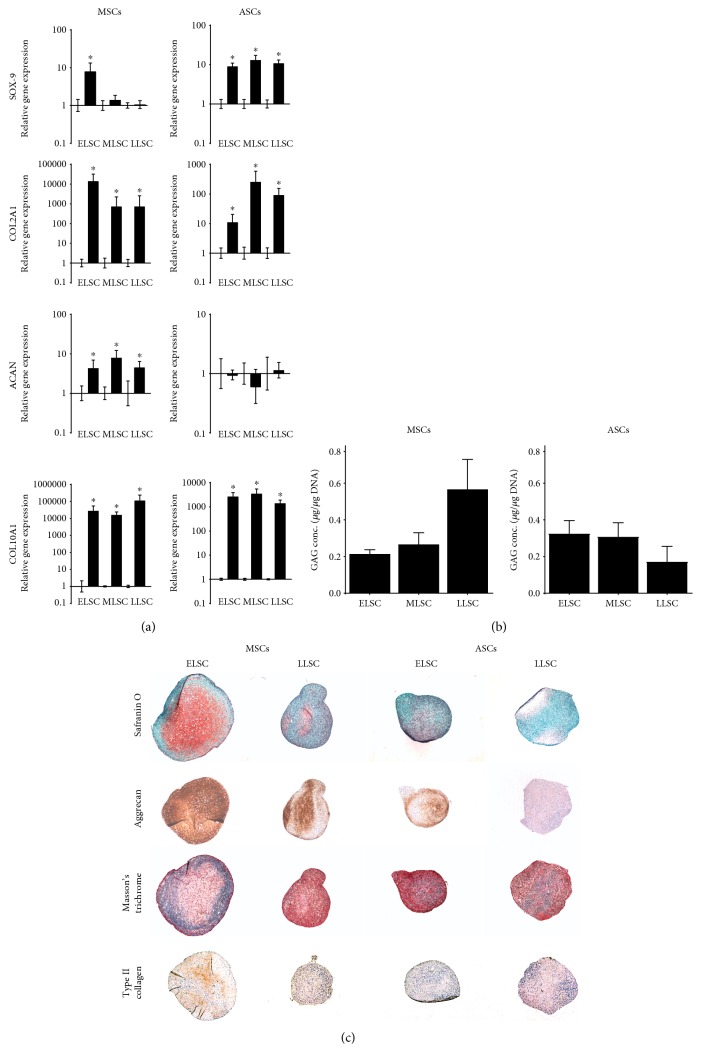
Assessment of the influence of monolayer expansion of the chondrogenic potential of donor-matched MSCs and ASCs. (a) Quantitative real-time PCR analysis of chondrogenic marker genes SOX-9, COL2A1, ACAN, and COL10A1 at ELSC, MLSC, and LLSC in MSCs and ASCs following pellet culture for 21 days in chondrogenic media. Data was normalised to the mean of the reference genes MRPL19 and EIF2B1 and presented as relative fold change in gene expression compared to day 0 controls plotted on a log scale. *N* = 5. Values are mean ± SEM. ∗ = *P* < 0.05. (b) DMMB analysis of GAG synthesis in MSCs and ASCs at ELSC and LLSC. Data represents mean GAG concentration from two pellets for each cell sample (*μ*g/*μ*g DNA) ± SEM. *N* = 5. ∗ = *P* < 0.05 compared to day 0 control. (c) Representative histological images of ELSC and LLSC in MSC and ASC pellets following 21 days in culture in a chondrogenic media. All images taken at equivalent magnification to allow comparison of size of pellets.

Chondrogenic differentiation in pellet cultures was evaluated by chondrogenic gene expression, GAG synthesis, histological staining with safranin O and Masson's trichrome, and immunohistochemistry for type II collagen and aggrecan. Expression levels of all chondrogenic marker genes were significantly upregulated in MSCs at ELSC following 21 days in chondrogenic media, while ASCs at ELSC demonstrated no change in aggrecan expression following pellet culture, but significant increases in SOX-9, COL2A1, and COL10A1. With increasing lifespan, MSCs retained differentiation capacity, demonstrating increased expression of COL2A1, ACAN, and COL10A1 at both MLSC and LLSC following pellet culture, but no significant increase in SOX-9 at either timepoint. ASCs likewise retained differentiation capacity, demonstrating significant increases in expression of SOX-9, COL2A1, and COL10A1 following 21 days of pellet culture in chondrogenic media at both MLSC and LLSC timepoints; however, interestingly aggrecan gene expression remained at baseline levels (Figure 4(a)). Extraction and quantification of GAGs from the pellets displayed no significant lifespan-dependent difference in the amount of GAG formation in MSCs and ASCs, and no significant difference in GAG levels were observed between cell types (Figure 4(b)). Histological staining at ELSC showed greater deposition of proteoglycan and collagen within MSCs compared to ASCs, with proteoglycan distribution throughout the pellet and collagen deposition largely located at the periphery of the cell pellet (Figure 4(c)). Extracellular matrix deposition within ASCs at ELSC appeared more randomly distributed, with collagen deposition often mirroring the deposition of proteoglycan. Histological staining for proteoglycan and collagen was less intense in MSCs at LLSC compared to ELSC, while ASCs demonstrated no obvious change in collagen deposition but a loss of proteoglycan deposition by LLSC. Immunohistochemistry confirmed expression of both aggrecan and type II collagen by MSCs, which was diminished by LLSC. ASCs demonstrated lower levels of aggrecan staining at both timepoints compared to MSCs and only weak staining for type II collagen at ELSC which was absent at LLSC.

#### 3.2.2. Osteogenic Differentiation

**Figure 5:**
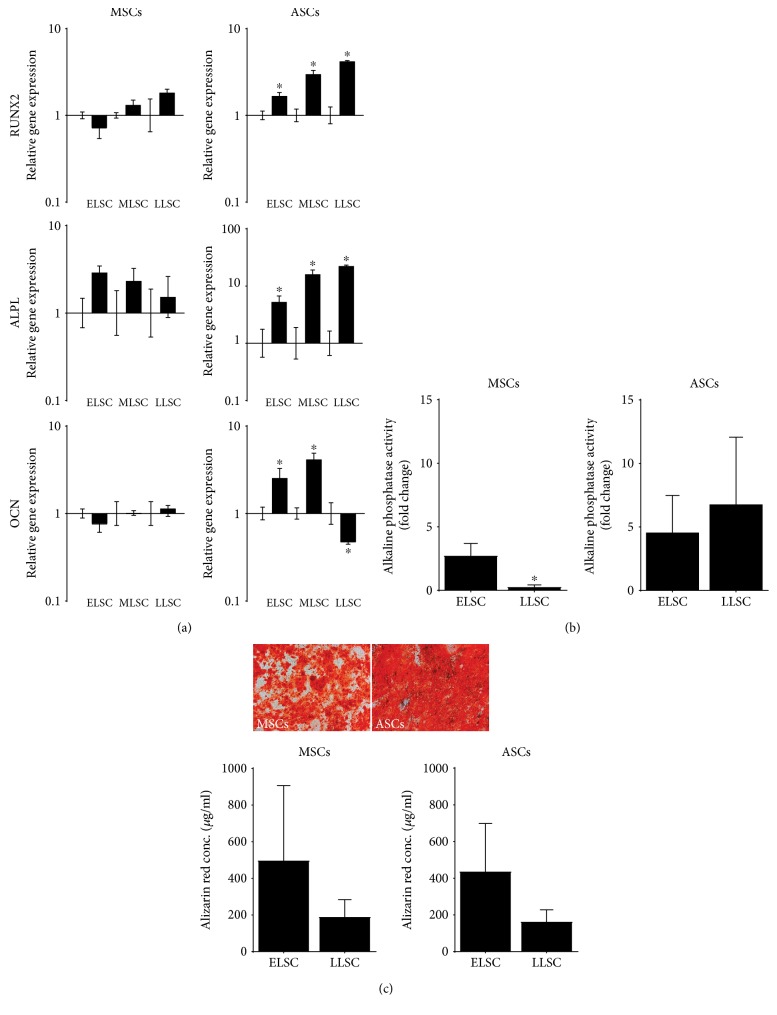
Assessment of the influence of monolayer expansion of the osteogenic potential of donor-matched MSCs and ASCs. (a) qPCR analysis of osteogenic marker at ELSC, MLSC, and LLSC in MSCs and ASCs following culture for 21 days in osteogenic media. Data was normalised to the mean of the reference genes MRPL19 and EIF2B1 and presented as relative fold change in gene expression compared to day 0 controls plotted on a log scale. *N* = 5. Values are mean ± SEM. ∗ = *P* < 0.05. (b) Quantification of alkaline phosphatase activity in MSCs and ASCs from triplicate wells at ELSC and LLSC. Data represent mean fold change in activity ± SEM compared to day 0 controls. *N* = 5. ∗ = *P* < 0.05. (c) Representative images of alizarin red staining of ELSC MSCs and ASCs following 0 days and 21 days in culture in osteogenic media and quantification of alizarin red staining in MSCs and ASCs at ELSC and LLSC. Data represent mean alizarin red concentration ± SEM. *N* = 5. ∗ = *P* < 0.05.

Osteogenic differentiation in monolayer culture was evaluated by osteogenic gene expression, alkaline phosphatase activity, and alizarin red S staining. While expression levels of osteogenic marker genes in MSCs did not appear to change following 21 days in osteogenic media at any lifespan stage, ASCs revealed significant upregulation in all genes at all lifespan stages, with the exception of osteocalcin at LLSC which displayed a significant decrease in expression (Figure 5(a)).

Alkaline phosphatase activity demonstrated similar increases following differentiation of both MSCs (2.7-fold ± 0.9) and ASCs (4.5-fold ± 2.9) at ELSC compared to day 0 controls. At LLSC MSCs demonstrated virtually no increase in alkaline phosphatase activity compared to day 0 controls (0.2-fold ± 0.1), whereas activity in ASCs increased 6.7-fold ± 5.3 (Figure 5(b)). Alizarin red S staining of MSCs typically revealed heavy staining across the monolayer of ASCs, while staining was lighter and patchy within MSC monolayer cultures (Figure 5(c)). Following extraction and quantification of the alizarin red S stain, no significant difference in alizarin red S concentration was identified between MSCs and ASCs. While levels appeared lower in both cell types at LLSC compared to ELSC, this difference was not significant.

#### 3.2.3. Adipogenic Differentiation

**Figure 6:**
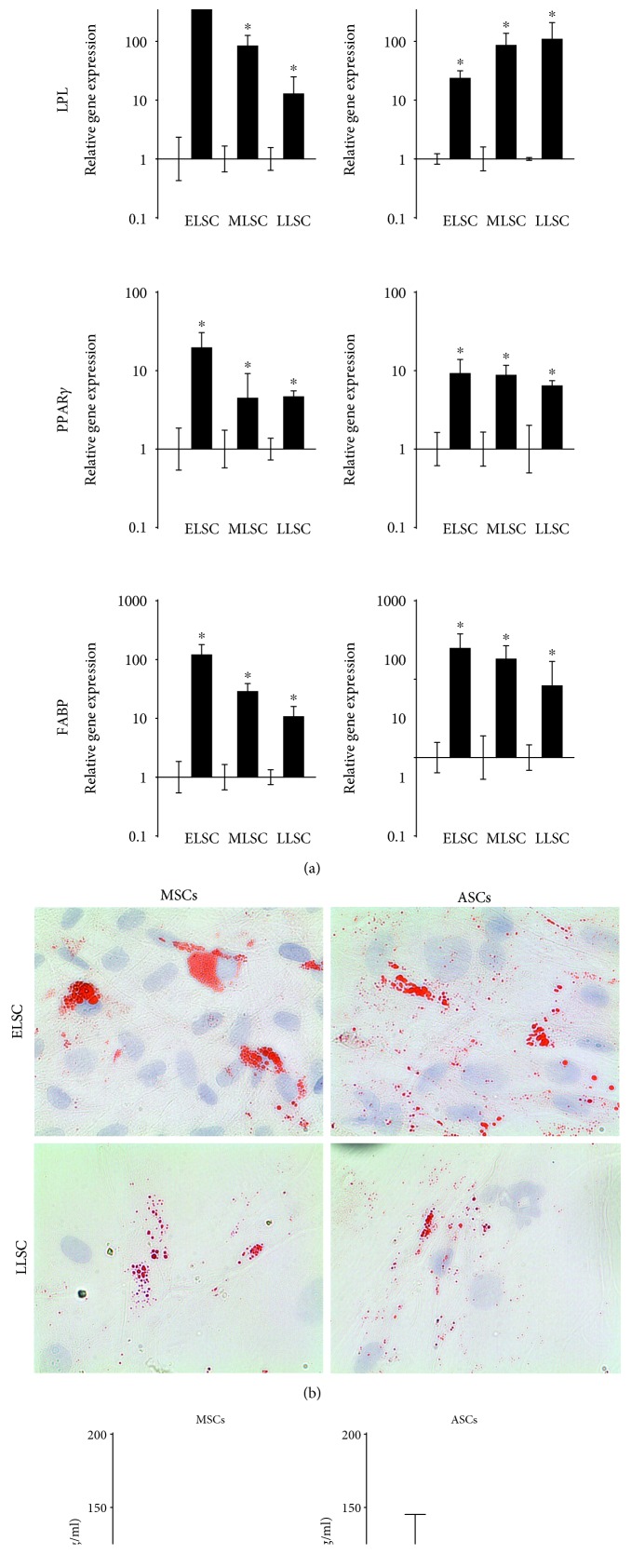
Assessment of the influence of monolayer expansion of the adipogenic potential of donor-matched MSCs and ASCs. (a) qPCR analysis of adipogenic marker genes at ELSC, MLSC, and LLSC in MSCs and ASCs following culture for 21 days in adipogenic media. Data was normalised to the mean of the reference genes MRPL19 and EIF2B1 and presented as relative fold change in gene expression compared to day 0 controls plotted on a log scale. *N* = 5. Values are mean ± SEM. ∗ = *P* < 0.05. (b) Representative images of oil red O staining in ELSC and LLSC MSCs and ASCs following 21 days in culture in adipogenic media. (c) Quantification of oil red O staining in MSCs and ASCs from triplicate wells at ELSC, MLSC, and LLSC. Data represents mean oil red O concentration (*μ*g/ml) ± SEM. *N* = 5.

Adipogenic differentiation was assessed by evaluating adipogenic gene expression and oil red O staining. Expression levels of adipogenic marker genes revealed significant upregulation following 21 days in adipogenic media for both MSCs and ASCs at ELSC, MLSC, and LLSC (Figure 6(a)). While increases in adipogenic gene expression by MSCs appeared to be diminished with increasing lifespan, this trend was not as clear in ASCs, with increases in LPL being larger at LLSC compared to either ELSC or MLSC. Oil red O staining of MSCs at ELSC typically revealed larger regions of dense staining within cells, while ASCs displayed multiple small staining patterns (Figure 6(b)). At the LLSC timepoint, ORO staining of both cell types was scarce throughout the cultures with regions of small stained droplets. Following extraction and quantification of the ORO stain, no significant difference in ORO concentration was identified between ELSC and LLSC for either cell type. However, ASCs appeared to have higher ORO concentration at ELSC than matched MSCs. This level dropped in ASCs by LLSC to levels similar to that shown by MSCs (Figure 6(c)).

## 4. Discussion

The regenerative potential of MSCs and ASCs as a cell therapy tool for a variety of age-related musculoskeletal disease is currently an area of great interest [5]. However, tissue engineering and regenerative medicine approaches often require large numbers of cells; therefore, given the relatively low frequency of MSCs within tissues, ex vivo expansion is unavoidable in order to obtain an appropriate number of cells for clinical treatment [43]. However, expansion in vitro rapidly ages cells and may therefore induce changes to their characteristics and functionality, rendering them less desirable for clinical use. Current studies report conflicting influences of in vitro expansion on MSC differentiation potential, and where studies have compared MSCs and ASCs, they routinely use cells from different donors. By using donor-matched cells, this study offers a unique insight into whether in vitro ageing induces a senescent cell phenotype or functional decline within MSCs and ASCs and whether both cells types are equally affected. The findings will therefore have implications on their relative applications in musculoskeletal tissue engineering and regenerative medicine therapies.

### 4.1. Comparison of Phenotype and In Vitro Proliferative Capacity between Donor-Matched MSCs and ASCs

Both MSCs and ASCs demonstrated equivalently high levels of CD73, CD90, and CD105 expression and low levels of classic negative markers, in line with published criteria [9, 10, 44], and interestingly, the expression of these markers did not appear to change substantially during in vitro expansion. Both MSCs and ASCs also demonstrated classic elongated morphology during early culture, but with time in culture, increasing numbers of both cell types displayed evidence of a senescent morphology [45, 46]. This change in morphology occurred alongside a decline in proliferation rate, suggesting senescence of both MSCs and ASCs with time in culture. However, importantly, ASCs expanded faster and reached a higher CPD than donor-matched MSCs. Several studies have reported a similar lifespan for human MSCs to that found here [11, 15, 47]. Conversely, the few studies investigating ASCs suggest that they can reach 34–44 CPDs [12, 37], well in excess of the numbers reached for any sample in this study. The lower CPD observed in ASCs in this study compared to previous studies may be due to isolation site and isolation procedure, since previous studies have reported a significantly higher frequency of ASCs within adipose tissue from the abdomen compared to the hip/thigh region and from liposuction material compared to excised tissue [48, 49].

The increased lifespan demonstrated by ASCs in comparison to donor-matched MSCs may be due, at least in part, to the significantly higher mean telomere length identified in ASCs, which was maintained throughout their lifespan. Interestingly, while MSCs demonstrated an insignificant decline in mean telomere length with time in culture, telomere length in ASCs decreased significantly between ELSC, MLSC, and LLSC, reflecting the increased proliferation in these cells. The reason for higher mean telomere length in ASCs compared to MSCs from the same donor is unclear; however, it may reflect a lower in vivo demand on ASCs compared to MSCs, although further studies would be required to confirm this.

Replicative senescence is characterised through a number of features including critical telomere shortening, as well as high levels of cell cycle proteins p16^ink4a^, p21, and p53 which are involved in regulating the cellular senescence process. Interestingly, while p21 and p53 demonstrated no significant differences either between cell type or with time in culture, p16^ink4a^ expression was significantly lower in ASCs compared to MSCs throughout replicative lifespan. The expression of p16^ink4a^ increased in both MSCs and ASC with time in culture, but the significantly lower expression in ASCs throughout culture further explains the enhanced proliferative capacity of these cells compared to MSCs. Interestingly, oxidative stress has been reported to increase p16^ink4a^ expression, with production of stress signalling molecules such as ROS from mitochondria during long-term culture leading to p16^ink4a^ [50]. A culture of MSCs under hypoxic conditions has been shown to downregulate p16^ink4a^ compared to a normoxic culture [51] and to enhance proliferation [47]; hence, expansion under hypoxic conditions may be one method to accelerate proliferation and reduce the detrimental increase in p16^ink4a^ expression.

The expression of Nanog has also been suggested to be involved in MSC lifespan through a number of mechanisms including upregulation of genes involved in the cell cycle, DNA replication, and DNA damage repair [52]. While expression of Nanog may reflect an adaptation to culture in vitro, rather than a native in vivo expression, a high level of expression is through to relate to increased lifespan and, confirming this hypothesis, forced expression of Nanog significantly increases the total number of PDs achieved before senescence in MSCs [33]. Therefore, while no significant difference in the expression of Nanog was observed with time in culture, the higher levels of Nanog expression identified in ASCs compared to MSCs throughout lifespan may be partially responsible for the enhanced proliferative capacity observed in ASCs.

Taken together, these data demonstrate that ASCs represent a population of cells with enhanced proliferative capacity compared to MSCs and therefore may produce the required numbers of cells for musculoskeletal tissue engineering and regenerative medicine applications more rapidly than MSCs.

### 4.2. Impact of In Vitro Expansion of Differentiation Capacity of Donor-Matched MSCs and ASCs

Previous studies suggest that while subpopulations of MSCs retain multipotentiality through a number of passages, most MSC cultures tend to progressively lose this capacity [14, 29]. Conversely, the multipotency of ASCs has been shown to be retained during long-term culture [36–38]; however, direct comparison of the osteogenic, chondrogenic, and adipogenic capacity of donor-matched human MSCs and ASCS has not been described.

MSCs maintained their chondrogenic capacity throughout lifespan and, while pellets appeared smaller at LLSC compared to ELSC, the gene expression and GAG data suggest appropriate differentiation and ECM synthesis even at LLSC. Donor-matched ASCs also demonstrated induction of chondrogenic marker gene expression irrespective of lifespan point. However, pellets appeared smaller than MSC pellets at all lifespan points and stained less intensely for both type II collagen and aggrecan, with aggrecan gene expression also not demonstrating upregulation in ASCs at any lifespan point. Previous studies on MSCs suggest either reduced efficiency or rapid loss in chondrogenic differentiation potential with time in culture [23, 29, 30, 33, 34], which was not identified here. Previous studies investigating the effect of long-term culture on ASC chondrogenic capacity demonstrated retention of chondrogenic differentiation potential [23, 36, 38], in line with findings reported here. However, the diminished aggrecan production compared to MSCs has previously been reported and may suggest that MSCs are the more appropriate choice for chondrogenic applications [21, 22]. However, differences in growth factor receptor expression have previously been shown to influence ASC response to TGF*β*, and hence, application of alternative growth factors such as BMP6 may restore functional matrix deposition [53], although further investigation would be required to elucidate this.

This study demonstrated a general decline in osteogenic capacity of MSCs with increasing time in culture, with the induction of osteogenic marker genes, alkaline phosphatase activity, and alizarin red concentration all being diminished at LLSC compared to ELSC. This agrees with previous studies which report a complete loss of osteogenic capacity with extended culture [14, 23, 30–32, 35]. Conversely, ASCs did not appear to lose osteogenic capacity with time in culture, with the induction of osteogenic marker genes, alkaline phosphatase activity, and alizarin red concentration all being similar at LLSC compared to ELSC. These findings also agree with earlier studies on ASCs, which also confirmed maintenance of osteogenic potential within ASCs over long-term culture [23, 38]. These data suggest that, for osteogenesis at least, ASCs may be an attractive proposition when compared to MSCs from the same donor.

Both MSCs and ASCs demonstrated capacity for adipogenic differentiation, with increases in marker gene expression increasing significantly following culture in adipogenic media, irrespective of lifespan point. ASCs demonstrated enhanced oil red O staining at ELSC compared to MSCs, but this difference was lost by LLSC, potentially suggesting a decline in function. Previous studies have also reported a negative effect of long-term culture on MSC adipogenic potential, describing either a decrease in the number of adipocytes formed in late passages compared with early, a delay in appearance of lipid vacuoles, or even a complete loss of adipogenic potential [14, 23, 29, 34, 45, 54]. Conversely, ASCs have previously been shown to retain adipogenic potential through extended culture [23, 37, 38]. Our study highlights the importance of functional assessments in determining differentiation capacity, since cells throughout lifespan were capable of upregulating adipogenic genes, but lipid vacuoles and oil red O staining appeared to be reduced with extended culture. The lower levels of staining observed in MSCs may also reflect the more senescent phenotype displayed by these cells during early culture, although more work is required to establish exactly which parameters of ageing are responsible for any potential loss of adipogenic potential.

## 5. Conclusions

This study has directly compared the proliferative capacity and multipotency of donor-matched MSCs and ASCs. The findings suggest that, while both cell types display diminished proliferative capacity with extended in vitro expansion, ASCs have a “younger” phenotype which permits enhanced proliferation before replicative senescence occurs. This is important for clinical application in musculoskeletal tissue engineering and regenerative medicine therapies where a large number of cells are required in as little time as possible.

As ASCs demonstrated an enhanced proliferative capacity and similar differentiation capacity to MSCs from the same donor, this study suggests that ASCs may be a preferable cell source for musculoskeletal tissue engineering and regenerative medicine applications, although further work would be needed to demonstrate how these two cell populations compare in vivo.

## Figures and Tables

**Table 1:** Primer and probe details: gene name, accession number, sequences of forward and reverse primers and probes, and primer concentrations.

Gene name	Accession number	Forward sequence	Reverse sequence	Probe sequence	Primer concentration (nM)
MRPL19	NM_014763	CCACATTCCAGAGTTCTA	CCGAGGATTATAAAGTTCAAA	CAAATCTCGACACCTTGTCCTTCG	900
EIF2B1	NM_001414	TCCCAGATAAGTTTAAGTATAAG	AGCAGAGTGATTAAGGAA	CGCAGACTGGACAAGACCTCA	600
p16^ink4a^	NM_000077	CCAGAGGCAGTAACCATG	GACCTTCGGTGACTGATG	AGGTCCCTCAGACATCCCCG	900
p21	NM_000389	CCAGCATGACAGATTTCTA	GAGACTAAGGCAGAAGATG	CGGCTGATCTTCTCCAAGAGGAA	450
p53	NM_000546	CTTGGAACTCAAGGATGC	TTTTATGGCGGGAGGTAG	TTTTGGACTTCAGGTGGCTGGA	450
Nanog	NM_02486	CCTGAAGAAAACTATCCATCC	AGAGGAGAGACAGTCTCC	CAAATGTCTTCTGCTGAGATGCCT	450
RUNX2	NM_001024630	CGCTGCAACAAGACC	CGCCATACAGTAACC	TGGCCTTCAAGGTGGTAGCCCTC	900
ALPL	NM_000478	ACGTCTTCACATTTGGTG	GGTAGTTGTTGTGAGCATA	ACTCTATCTTTGGTCTGGCCCC	450
OCN	NM_199173	CCGCACTTTGCATCG	GCCATTGATACAGGTAGC	CCAGGCAGGTGCGAAGCCC	600
PPAR*γ*	NM_005037	TCTCCACCTTATTATTCTGA	CTGTCATAGATAAGCTTCAA	CGGAAGAAACCCTTGCATCCTT	450
Adipsin	NM_001928	GAAGGTGCAGGTTCTC	CAGGGCCCAGTGTG	TCTCCGACAGCTGTAGCAG	450
LPL	NM_000237	AGGTGATCTTCTGTTCTAG	GCCGTTCTTTGTTCTG	CGCCCAGTTTCAGCCTGAC	600
FABP4	NM_001442	CTGGTACATGTGCAGAAA	ATCCCACAGAATGTTGTA	TCA[+G]TC[+C]AG[+G]TC[+A]ACG	900
SOX-9	NM_000346	GCTCTGGAGACTTCTGAA	GGTACTTGTAATCCGGGTG	TCCTCCTTGTGCTGCACGCG	450
COL2A1	NM_033150	CAGTGGTAGGTGATGTTC	GGCTTCCATTTCAGCTATG	CCAACACTGCCAACGTCCAG	450
ACAN	NM_001135	GGCTTCCACCAGTGTGAC	GTGTCTCGGATGCCATACG	TGACCAGACTGTCAGATACCCCATCCA	900
